# National Monitoring of Veterinary-Dispensed Antimicrobials for Use on Pig Farms in Austria: 2015–2020

**DOI:** 10.3390/antibiotics11020216

**Published:** 2022-02-08

**Authors:** Clair L. Firth, Reinhard Fuchs, Klemens Fuchs

**Affiliations:** 1 Unit of Veterinary Public Health & Epidemiology, University of Veterinary Medicine, 1210 Vienna, Austria; clair.firth@vetmeduni.ac.at; 2 Data, Statistics and Risk Assessment, Austrian Agency for Health and Food Safety (AGES), 8010 Graz, Austria; reinhard.fuchs@ages.at

**Keywords:** antimicrobial use, pigs, veterinary, monitoring

## Abstract

Antimicrobial use in livestock production systems is increasingly scrutinised by consumers, stakeholders, and the veterinary profession. In Austria, veterinarians dispensing antimicrobials for use in food-producing animals have been required to report these drugs since 2015. Here, we describe the national monitoring systems and the results obtained for Austrian pig production over a six-year period. Antimicrobial dispensing is described using the mass-based metric, milligrams per population correction unit (mg/PCU) and the dose-based metric, Defined Daily Dose (DDDvet) per year and divided into the European Medicines Agency’s prudent use categories. Pig production was divided into breeding units, fattening farms, farrow-to-finish farms, and piglet-rearing systems. Over all six years and all pig production systems, the mean amount of antimicrobials dispensed was 71.6 mg/PCU or 2.2 DDDvet per year. Piglet-rearing systems were found to have the highest levels of antimicrobial dispensing in DDDvet, as well as the largest proportion of Category B antimicrobials, including polymyxins. Although progress has been made in promoting a more prudent use of antimicrobials in veterinary medicine in Austria, further steps need to be taken to proactively improve animal health and prevent disease to reduce the need for antimicrobials, particularly those critically important for human medicine, in the future.

## 1. Introduction

Globally, antimicrobial use in agriculture, particularly in food-producing animals, is increasingly seen critically by consumers [1]. Although the use of antimicrobials as growth promoters has been banned in the European Union (EU) since 2006, these medications are often still used for disease prophylaxis, and reductions in antimicrobial use (AMU) are both possible and necessary in order to ensure their continued effectiveness against bacterial infections. From 2022, the new Veterinary Medicinal Products Regulation (2019/6) in the EU will legislate new restrictions on AMU in veterinary medicine and requires all member states to monitor and record veterinary AMU in their countries, initially in food-producing animals, but eventually (from 2029) in pets as well [2].

The excessive use of antimicrobials in pig production initially came under criticism in Denmark in the 1990s, and the country was among the first to successively ban a variety of antimicrobials as growth promoters from 1995 onwards [1,3]. Denmark also led the way in benchmarking pig producers and introducing penalty schemes, such as the yellow card for excessive antimicrobial use in 2010 [3]. A number of other European countries, such as the Netherlands, also began to document their veterinary antimicrobial use, and the first EU report of veterinary antimicrobial sales (the ESVAC report) was published by the European Medicines Agency in 2011, using sales data from nine countries [4].

The Austrian health authorities began contributing data on veterinary antimicrobial sales from pharmaceutical companies/wholesale pharmacies to the European Union’s annual ESVAC report in 2010. To date, reported sales of veterinary antimicrobial drugs in Austria for food-producing animals have ranged from a maximum of 63 mg/population correction unit (PCU) in 2010 to a minimum of 42.6 mg/PCU in 2019 [5,6]. Since 2015, it has been required by local law for all veterinarians in Austria who dispense antimicrobials for use in food-producing animals to annually report the amounts dispensed to the relevant authorities [7]. In addition, antimicrobials are only available from veterinarians, and, since 2005, injectable (as well as intramammary and intrauterine) antimicrobials have been further restricted and can only be dispensed to farmers who are members of the Austrian Animal Health Service (*Tiergesundheitsdienst*, TGD) and have completed training courses in the use and administration of veterinary medications [8,9]. Antimicrobials administered directly by the veterinarians themselves do not currently have to be reported, although their use is documented in both veterinary practice and on-farm records [7].

Pig production in Austria is not an extremely large industry, when compared internationally. The average herd size is 133 head (ranging from 15,950 holdings keeping only 1–3 pigs to 12 units with more than 3000 pigs) [10,11]. Based on official data available with respect to the reference day of 1st June each year, the pig population included here ranged from a minimum of 2,773,225 pigs in 2019 to a maximum of 2,845,451 in 2015 (mean number of pigs from 2015–2020: 2,802,433; median: 2,799,632) [10]. Pig producers are primarily located in the federal states of Upper Austria (39.8% of total pig numbers in 2020), Lower Austria (27.0%), and Styria (26.8%) [10].

The most recent national data records in metric tonnes in 2020 reported that 73.4% of all veterinary antimicrobials dispensed in Austria were for use in pigs (ranging from 71.8–76.4% between 2016 to 2020), compared to 19.7% in cattle (beef and dairy) and 6.7% in poultry [12]. However, when comparing these figures to other countries, it is important to note that the Austrian national-monitoring system currently only includes antimicrobials dispensed by veterinarians to farmers and does not include those administered by the veterinarians themselves.

The data presented here represent the results of the national monitoring of veterinary antimicrobials dispensed between 2015 and 2020. To allow comparison with other countries and systems, the data analysis focuses on using international metrics, such as mg/PCU (population corrected unit) and Defined Daily Doses (DDDvet), as published by the European Medicines Agency and recommended by European expert groups [13,14].

## 2. Results

### 2.1. Study Population

**Figure 1:**
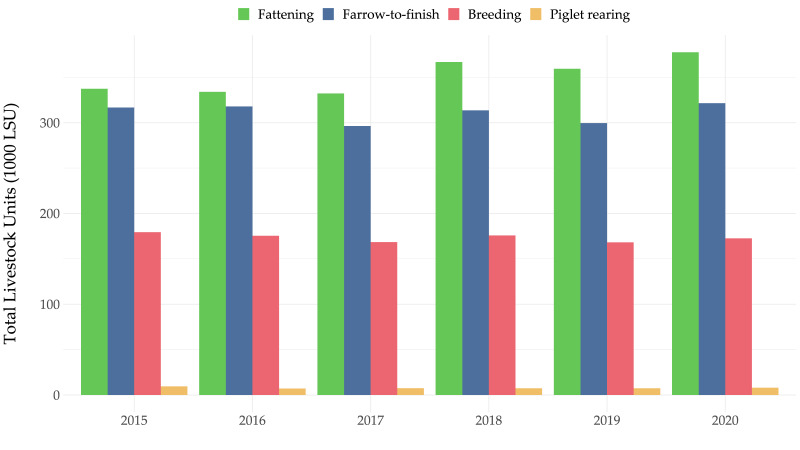
Comparative numbers of 1000 livestock units (LSU) in Austrian pig production systems (included in the study population, i.e., farms where antimicrobials were dispensed and reported) between 2015 and 2020.

Pig production in Austria is divided into farrow-to-finish farms, fattening farms, breeding units, and piglet-rearing units. Figure 1 shows the proportions of the different pig production systems in the study population over the years included here. The study population (i.e., farms where antimicrobials were dispensed and reported to the authorities by herd veterinarians) covers between 81% (in 2015) and 87% (in 2020) of the total national pig production.

Data are provided in standardised livestock units, as defined by the Austrian Ministry of Agriculture [15]. The vast majority of pigs included in this study population were kept in fattening and farrow-to-finish units (mean: 351,261 and 310,933 LSU; median 348,398 and 315,147 LSU, respectively). An extremely small number of pigs are reared in piglet-rearing systems (mean: 7809 LSU; median 7450 LSU) (Figure 1).

### 2.2. Overall Antimicrobials Dispensed

#### 2.2.1. Mass-Based Metrics (mg/PCU)

All veterinarians treating farm animals and dispensing antimicrobials to farmers for use in such animals are required by Austrian law to report their annual dispensed amounts [7]. The data included here are taken from these national records of annual antimicrobial monitoring between 2015 and 2020 [12].

**Figure 2:**
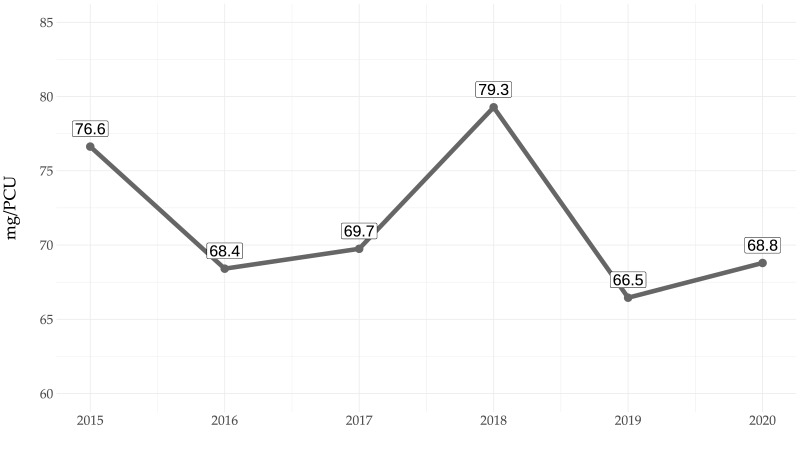
Amount of antimicrobials (mg/PCU) dispensed for use in pigs in Austria between 2015 and 2020.

Antimicrobials dispensed by herd veterinarians for use in pig production between 2015 and 2020 are shown in milligrams per population correction unit (mg/PCU, as defined in the European Medicines Agency’s ESVAC report and calculated for the entire national pig herd [6]) in Figure 2. (NB. 1 PCU is approximately equivalent to 1 kg livestock biomass). For all pig production systems overall, the antimicrobial use ranged from a maximum of 79.3 mg/PCU in 2018 to a minimum of 66.5 mg/PCU in 2019.

Table 1 shows the proportions of antimicrobial dispensing by veterinarians for use in the various pig production systems. The vast majority of antimicrobial dispensing in mg/PCU over all six years was for use in farrow-to-finish and fattening farms. It is important to note that the decreasing proportion of pig production units that were “not assignable” to a specific production system has fallen dramatically (from 4.6% to 0.8%) since the monitoring system was first initiated in 2015. This is primarily due to improvements to the electronic-reporting system and the data-plausibility checks now in place.

#### 2.2.2. Comparison of Mass-Based and Dose-Based Metrics

A variety of antimicrobial monitoring guidelines and recommendations suggests the use of dose-based metrics, such as the European Medicines Agency’s DDDvet, to allow for divergences in dosing to be accounted for within AMU records [14,16]. Recording antimicrobial dispensing in mg/PCU often leads to an overestimation of some antimicrobials and an underestimation of others [16,17].

**Figure 3:**
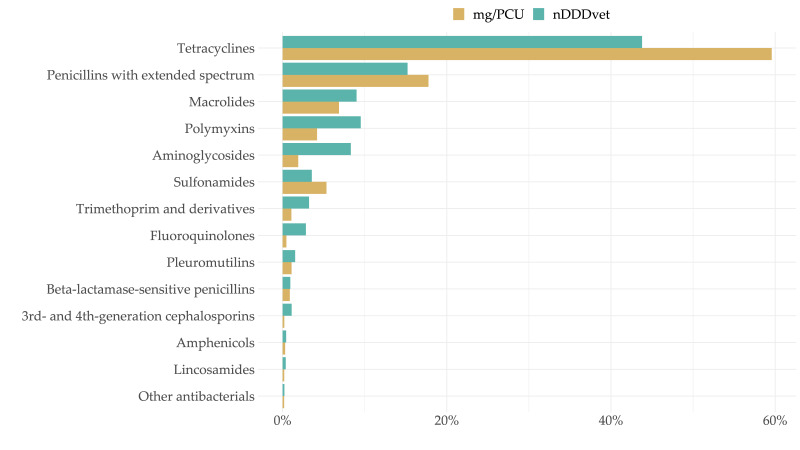
Comparison of the mean proportions of mass-based and dose-based metrics for antimicrobial classes dispensed for use in pigs in Austria in 2020.

Figure 3 demonstrates the proportions of the total antimicrobial-dispensing data collected in 2020 when analysed by mg/PCU or DDDvet. The differences between tetracyclines in mg/PCU (59.6% of all antimicrobials dispensed) compared to around 43.8% of all dispensed DDDvet are particularly striking. By contrast, aminoglycosides make up 8.3% of antimicrobials dispensed by DDDvet compared to just 1.9% by mg/PCU, and polymyxins make up a much higher proportion of overall use (9.5%) by DDDvet compared to under 5% as mg/PCU (Figure 3).

When antimicrobial dispensing is presented by the proportion of DDDvet per year for the entire monitoring period (see Table 2), tetracyclines continue to make up the largest proportion each year (ranging from a maximum of 50.43% in 2018 to a minimum 39.77% in 2019). Extended-spectrum penicillins make up a much lower proportion (between 13.72–15.54%) and remain in second place over the study period, while polymyxins and macrolides alternate for the third most frequently dispensed antimicrobials. By contrast, when antimicrobial dispensing is presented by proportion of mg/PCU, although tetracyclines continue to make up the vast majority of antimicrobial use (generally > 60%), polymyxins have fallen to fifth place and make up only 2.77% to 4.19% of antimicrobial dispensing (compared to a much higher proportion of between 6.93–9.51% when analysed by DDDvet/year) (Table 3).

### 2.3. Antimicrobials of Critical Importance to Human Medicine

Antimicrobial dispensing presented here is divided into categories as defined by the European Medicines Agency’s Antimicrobial Expert Group (AMEG) [18,19]. Category A is not included, as antimicrobials in this category are not licensed for use in veterinary medicine in the EU (although they may be used off-label in nonfood-producing animal species). Categories B and C are critically important for human medicine and should be used restrictively (Category B: 3rd and 4th generation cephalosporins, fluoroquinolones, and polymyxins) or with caution (Category C includes e.g., macrolides, extended-spectrum penicillins, amongst others). Category D antimicrobials should be used prudently and include tetracyclines, sulfonamide/trimethoprim, beta-lactamase-sensitive penicillins, etc. With the exception of macrolides, Category B (“restrict”) antimicrobials are comparable to the WHO’s highest-priority, critically important antimicrobials (HPCIA) [18,19]. Further details are provided in the Section 5.

**Figure 4:**
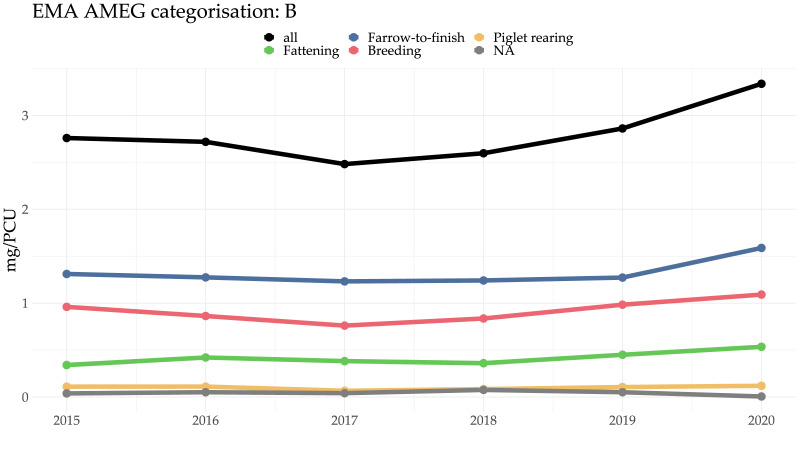
Antimicrobials (in mg/PCU) dispensed in Category B (the use of which should be “restricted”) by pig production system over time.

**Figure 6:**
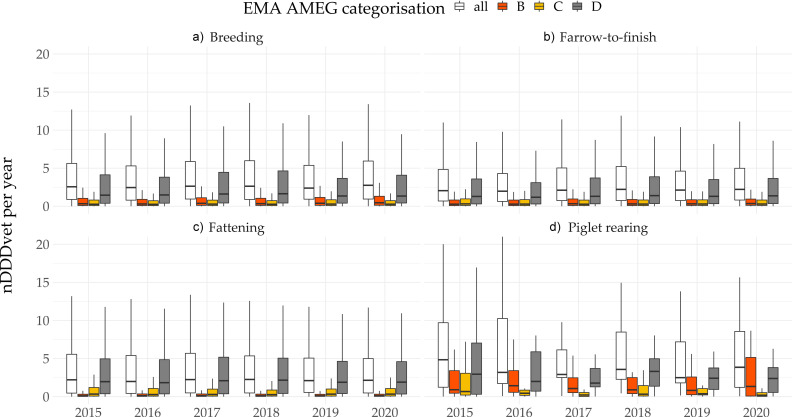
National recording of antimicrobials dispensed for use on a variety of pig production systems ((**a**) breeding units; (**b**) farrow-to-finish farms; (**c**) fattening farms; (**d**) piglet-rearing units) by European Medicines Agency antimicrobial category (B, C, D) and DDDvet/year.

In all production systems, the majority of antimicrobials dispensed were in Category D, with the exception of piglet-rearing units, where a substantial proportion of antimicrobials dispensed were in Category B. For details, see Figure 4 and Section 2.5 and Section 2.8 below. Again, the differences in mass-based versus dose-based metrics became apparent and can be seen very clearly when comparing Figure 4 (mg/PCU) with Figure 6d (DDDvet for piglet-rearing systems).

### 2.4. Route of Administration for the Dispensed Antimicrobials

**Figure 5:**
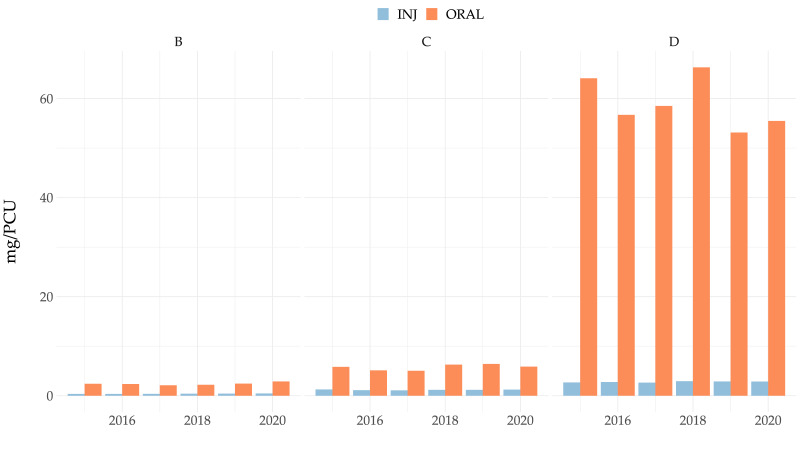
Antimicrobials (in mg/PCU) dispensed and divided by route of administration over time. INJ = systemic/injectable administration; ORAL = oral administration.

As would be expected, the vast majority of antimicrobials dispensed in all categories for use in Austrian pig production were for oral administration. Category D antimicrobials for oral use ranged from 53 mg/PCU in 2019 to around 66 mg/PCU in 2018, as shown in Figure 5. By mg/PCU, the most frequently dispensed antimicrobial class for oral use in Category D (“prudent use”) were tetracyclines (37.2 mg/PCU (2019)–50.8 mg/PCU (2018)) followed by macrolides (4.0 mg/PCU (2016)–5.2 mg/PCU (2018)) in Category C (“use with caution”) and polymyxins (2.1 mg/PCU (2017)–2.9 mg/PCU (2020)) (“restrict use”) in Category B; for details see Supplementary Figure S1. Injectable antimicrobials were dispensed at very low levels ranging from 0.4–0.5 mg/PCU in Category B to 2.6–2.9 mg/PCU in Category D (Figure 5). Specifically, the most commonly dispensed injectable antimicrobials were found in the classes of aminoglycosides (0.60 mg/PCU (2016)–0.75 mg/PCU (2015), Category C), beta-lactamase-sensitive pencillins (0.54 mg/PCU (2017)–0.60 mg/PCU (2020), Category D), and macrolides (0.27 mg/PCU (2019)–0.31 mg/PCU (2020), Category C), amongst others (Supplementary Figure S1).

### 2.5. Antimicrobial Use on Piglet production/Breeding Units

Breeding (piglet production) units made up approximately 20.5% of pig-producing units in Austria from 2015–2020, on average, ranging from 19.6% to 21.3% of pig production by LSU. Antimicrobial use on breeding pig units is shown in Figure 6a. The mean number of pigs kept on breeding units was 173,251 LSU.

### 2.6. Antimicrobial Use on Farrow-To-Finish Farms

Farrow-to-finish farms made up approximately 36.9% of pig-producing units in Austria from 2015–2020, on average, ranging from 35.9% to 38.1% of pig production by LSU (a mean number of pig equivalent to 310,933 LSU). Antimicrobial use on farrow-to-finish farms is shown in Figure 6b

### 2.7. Antimicrobial Use on Fattening Farms

Fattening/finishing farms made up approximately 41.7% of pig-producing units in Austria from 2015–2020, on average, ranging from 40.0% to 43.1% of pig production by LSU. The mean number of pigs kept on Austrian fattening farms was 351,261 LSU. Antimicrobial use on fattening farms is shown in Figure 6c, divided by EMA category. The vast majority of antimicrobials dispensed for use on fattening farms fall into Category D (prudent use).

### 2.8. Antimicrobial Use on Piglet-Rearing Farms

Piglet-rearing farms made up only a very small proportion of Austrian pig production, with, on average, approximately 0.9% of pigs produced in Austria from 2015–2020 by LSU (with a mean number of pigs equivalent to 7809 LSU). Only 23.3 of such farms reported antimicrobial use to the authorities, on average, over the six-year period (median 23.5 farms). Antimicrobial use on piglet-rearing units is shown in Figure 6d. It is important to note that the antimicrobial use in DDDvet per year on piglet-rearing farms is substantially higher than in other production systems. Furthermore, antimicrobial use in Category B (antimicrobials which are critical for human medicine and should be avoided) increased in 2020 to the highest level recorded since 2016 (median: 1.44 DDDvet/year in 2016 compared to 1.34 DDDvet/year in 2020).

## 3. Discussion

The data presented here provide a comprehensive overview of veterinary antimicrobial dispensing for use on Austrian pig farms over a six-year period. Given the mandatory nature of reporting and the fact that data were provided for between 81–87% of national pig production in Austria, these analyses can be considered an accurate representation of antimicrobial dispensing for use in pig production in the country. Nevertheless, it is important to note that antimicrobials administered directly by veterinarians themselves (rather than dispensed to farmers), while no doubt making up a small proportion of antimicrobial use in pig production overall, were not included in this dataset.

The most recent data available on total antimicrobial dispensing for all pig production systems in Austria were calculated to be 68.8 mg/PCU. (NB. 1 PCU is approximately equivalent to 1 kg livestock biomass). These figures are comparable with antimicrobial sales reported in a study of veterinary wholesale data in Switzerland in 2015 (77.4 mg/kg) [20], but are higher than those previously reported for a small convenience sample of 75 pig farms in Austria (mean over four years: 33.9 mg/kg) [21]. By contrast, the Austrian national figures are much lower than those recently reported for 67 Irish pig farms (161.9 mg/PCU) or the UK figures for the national pig herd in 2020 (namely 105 mg/kg) [22,23].

With respect to Defined Daily Doses (DDDvet), the mean value of the six-year median DDDvet per year (2.2 DDDvet/year) reported here and covering all pig production systems is difficult to compare with other dose-based metrics, as calculation methods vary. A recent study in Italy (using national DDD metrics) reported annual median values of between 6.24–7.57 DDDita/100 kg on 36 fattening farms [24], which is substantially higher than the Austrian national mean of the six-year median value of 2.17 DDDvet for fattening farms determined here. Meanwhile, a Swiss study of 227 pig farms reported a mean treatment of 4 DDDvet over a one-year period [25], which is also higher than that reported here in Austria.

When analysing antimicrobial use by substance, the Austrian data show that tetracyclines are dispensed in the greatest volumes by mass. However, it is important to note that mass-based calculations are often skewed with respect to older antimicrobial molecules which have higher dosage requirements in mg/kg than other newer drugs which may be more potent [14,16,26]. Oxytetracycline, for example, is licensed for use in pigs in Austria at a dosage of 40 mg/kg/d, which leads to a requirement of 2000 mg per day for a 50 kg pig. In contrast, the polymyxin, colistin, licensed at a dosage of 5 mg/kg/d, leads to a requirement of 250 mg per day for the same pig. This means that when comparing these antimicrobial drugs using mass-based metrics, oxytetracycline appears to be used at an eight-fold higher amount than colistin, which skews the overall proportions of antimicrobial classes in mg/kg. These discrepancies can be balanced out by using the defined daily dose (DDDvet), which refers to the daily dose as a whole, regardless of the amount of antimicrobial drug administered in milligrams.

For this reason, a comparison using dose-based metrics is essential [16]. Nevertheless, even when analysed by DDDvet metrics, tetracyclines still made up the majority (>55%) of antimicrobials dispensed for use in pig production in Austria between 2015–2020. Other studies have also reported that tetracyclines and penicillins are the most commonly used antimicrobials in pig production, such as a systematic review of 36 international papers [27] and a survey of 36 finishing pig farms in Italy [24]. In 2016, an Irish study of 67 farms, as well as Danish national reporting data, both demonstrated that tetracyclines were most frequently used [22,28], and similar findings have also been reported more recently from Japan [29]. The vast majority of tetracycline use in all these studies, as well as in the Austrian data presented here, was for oral administration. Whilst we do not have access to diagnoses data in Austria, tetracyclines are known to be commonly used for the treatment of gastrointestinal disorders and respiratory disease in pigs of all ages. Although tetracyclines are categorised by the EMA as the lowest level of caution (Category D, prudent use), some countries, such as Denmark, have seen increasing levels of antimicrobial resistance to this antibiotic and are now taking measures to reduce its routine use in pigs [28,30]. Similar resistance patterns have also been reported in studies in Austria, where tetracycline resistance was reported among 66% of *Streptococcus suis* isolates (increasing up to 88% of *Sc. suis* isolates obtained from joints) and 67.7% of *Escherichia coli* isolates obtained from piglets with diarrhoea [31,32].

Among piglet-rearing (and, to a much lesser extent, breeding) farms, a large proportion of antimicrobial dispensing was made up of polymyxins. This antimicrobial class contains the drug, colistin, which is commonly used to treat gastrointestinal disorders in young piglets (both pre- and post-weaning age), particularly disease caused by enterotoxigenic *Escherichia coli* (ETEC). While it is important to note that piglet-rearing farms make up only a very small proportion of Austrian pig producers (namely a mean number of pigs equivalent to 7809 LSU and between 0.8–1.5% of total antimicrobials dispensed for use in pigs by mg/PCU), polymyxins still made up a relatively large proportion (up to 9% by DDDvet, the third most frequently dispensed class in 2020) of antimicrobials dispensed in Austria overall. Polymyxins are classified by the European Medicines Agency as Category B antimicrobials, the use of which should be restricted as much as possible. Some countries, such as the UK and Denmark, have recently managed to avoid their use altogether among pig producers [23,28]. Although the most recent European Sales of Veterinary Antimicrobial Agents (ESVAC) report in 2021 stated that polymyxin use had fallen by 77% in 31 European countries since 2011, they are still sold at a higher level (based on mg/PCU metrics) in Germany, Poland, Hungary, Portugal, and Cyprus than in Austria [6]. The Netherlands has also reported a 7.3% increase in the use of colistin in all livestock production in 2020 and, as seen in the Austrian data, the vast majority of this use (91% of pig use) was for weaners [33]. Since plasmid-mediated colistin resistance was first detected in China in 2013, and the subsequent discovery of this resistance gene among pigs and humans throughout the world, recommendations have been made to reduce the use of this antimicrobial in livestock production wherever possible [34,35,36].

As would be expected, and as reported in many other studies [27,37,38], given the primarily intensive nature of pig production, the vast majority of antimicrobials were dispensed for oral administration. Systemically administered antimicrobials are generally used for the treatment of individual animals rather than entire groups and were dispensed at a very low level. Category D antimicrobials made up the largest proportion of antimicrobials dispensed for use by injection, namely 2.6 to 2.9 mg/PCU (compared to 53–66 mg/PCU for oral use). While dispensed at a much lower level than Category D antimicrobials for oral administration, Category B antimicrobials (including colistin) were more commonly dispensed for oral rather than systemic treatment, which is particularly concerning as a previous Austrian study of 75 pig farms demonstrated that oral treatments are frequently (in 75% of cases) underdosed and only 8% of cases were correctly dosed [21]. Furthermore, a number of studies have reported that the risk of antimicrobial resistance is substantially higher following oral antimicrobial treatment rather than parenteral administration of such drugs, and the European Medicines Agency also classes oral treatment, particular as a group treatment, to be the least preferable route of antimicrobial administration [19,39].

The data presented here have demonstrated that antimicrobials dispensed for use on pig units with a high number of young piglets make up the highest proportion of Category B antimicrobials, drugs which should be limited to restricted use. Here, it is particularly important for herd veterinarians to work together with pig producers to attempt to prevent disease, such as post-weaning diarrhoea, by improving hygiene and biosecurity, reducing stress, and vaccinating either breeding sows or young piglets whenever possible [40]. Given that colistin is critically important for human health (as the first-line drug for carbapenemase-producing *Enterobacteriaceae* infections), is primarily administered orally to pigs, and colistin-resistant bacteria have been isolated from wastewater from pig slaughterhouses in Germany, the use of this antimicrobial substance is an extremely relevant example of an essential One Health drug affecting human, animal, and environmental health [36,41,42]. For this reason, Austrian pig producers should attempt to learn from pig producers in other countries, where the use of colistin has been considerably reduced or stopped completely.

The implementation of the new EU Regulation 2019/6 will bring a number of changes to the use of veterinary antimicrobials in Austrian and European livestock production as a whole. Prophylactic use of antimicrobials will no longer be permitted, and only the metaphylaxis of a group will be allowed when one or more animal is proven to be infected. It is expected that the restrictions on the use of Category B antimicrobials will be tightened and enforced. For this reason, Austrian pig producers and their herd veterinarians will need to alter their antimicrobial use towards a more prudent use of these essential drugs in the future.

## 4. Conclusions

Based on mandatory veterinary reporting, antimicrobial dispensing in the pig sector in Austria has not decreased over the past six-year period. While the vast majority of antimicrobials dispensed are in the EU’s least restrictive Category D, an alarming proportion of Category B antimicrobials (primarily polymyxins, namely colistin) are dispensed for use in young piglets. National-benchmarking schemes are already in place for herd veterinarians and are currently being rolled out to individual pig producers. In future, partly due to new EU legislation, changes will need to be made to improve pig health and prudent antimicrobial use in this sector.

## 5. Materials and Methods

In Austria, pharmaceutical companies, marketing authorisation holders (distributors), and pharmaceutical wholesalers are required by law to provide the authorities with details of the sales of veterinary drugs containing antimicrobials. Additionally, veterinarians with in-house pharmacies must also report the quantities of antibiotics that are dispensed for use in food-producing animals for each farm and livestock species. The legal basis for the collection of these data is the “Veterinary Antibiotics Volumetric Flows Regulation” (*Veterinär-Antibiotika Mengenströme Verordnung*), which was enacted in 2014 [7].

### 5.1. Pig Population Data

The number of animals reared on each farm, as well as animal movement and official veterinary authority data, and numbers of animals slaughtered were available from the official veterinary database, namely the “Veterinary Information System (VIS)”.

Each farm was categorised into one farm type using the reported “production system type”, and the number of pigs in each category (piglets, fattening pigs, breeding sows/boars) are from the VIS database. The categorisation was taken from official records and can broadly be defined as follows. Breeding units refers to farms where sows (and sometimes boars) are kept to produce piglets for sale (it is not known at the veterinary authority level whether these piglets then go on to fattening farms or piglet rearing units). Fattening farms rear grower/finisher pigs from 20–32 kg liveweight up to slaughter. Piglet-rearing units keep piglets from weaning (i.e., the sows are not present on this type of farm) until the beginning of the fattening period (approx. 20–32 kg). As the name would suggest, farrow-to-finish farms rear piglets from birth to slaughter.

### 5.2. Antimicrobial Use Data

Veterinarians with in-house practice pharmacies are required to provide the amount of dispensed antimicrobials for each marketing authorisation identification number (i.e., each licensed pharmaceutical product) for each farm and livestock species. This is used to calculate the total metric tonnes dispensed of each antimicrobial active ingredient each year. This metric was then converted into mg/PCU for pigs using the standardised method used by the Austrian authorities for the entire national pig herd and described for national reporting for the European Union’s ESVAC report [6]. The standardised weight of a slaughtered pig as part of the PCU calculation is 65 kg; further details on the calculation of the PCU are provided elsewhere [6].

Furthermore, the number of Defined Daily Doses (DDDvet) for each antimicrobial substance, as defined by the European Medicines Agency, for the treatment of pigs was calculated as follows. The total number of milligrams of active ingredient dispensed for each antimicrobial substance was divided by the number of DDDvet for that antimicrobial substance with respect to pigs and the route of administration [13] to obtain the potential total number of Defined Daily Doses (DDDvet) for 1 kg animal biomass. To calculate the number of DDDvet per year, the following formula was used:

$$\mathrm{DDDvet}\;\mathrm{per}\;\mathrm{year}\;=\;\frac{\mathrm{Total}\;\mathrm{annual}\;\mathrm{number}\;\mathrm{of}\;\mathrm{DDDvet}\;\mathrm{per}\;1\;\mathrm{kg}\;\mathrm{biomass}}{\mathrm{Herd}\;\mathrm{size}\;\mathrm{of}\;\mathrm{breeding}\;\mathrm{animals}\;\left(\mathrm{if}\;\mathrm{present}\right)\;+\;\mathrm{No}.\;\mathrm{animals}\;\mathrm{moved}/\mathrm{slaughtered}\;\left(\mathrm{in}\;\mathrm{kg}\right)\;\mathrm{for}\;\mathrm{that}\;\mathrm{year}}$$

Livestock numbers were estimated based on the number of reported animals on the farm combined with animal movement and slaughter data. To ensure uniformity, livestock numbers were converted into the Austrian Ministry of Agriculture’s livestock units (LSU), e.g., piglets and weaners (up to 20 kg liveweight) are classified as 0.07 LSU, growers and young boars/sows (up to 50 kg liveweight) as 0.15 LSU, and breeding boars/sows as 0.30 LSU [15].

The data were also divided by route of drug administration, such as systemic or oral application, as well as by production group.

### 5.3. Classification into Prudent Use Categories

In addition, data were divided into groups based on the European Medicines Agency’s classifications of B (restrict use), C (use with caution), and D (use prudently), as well as according to the World Health Organization category of “highest priority critically important antimicrobials” (HPCIAs) [18,43]. For details, see Table 4. (NB. The EMA classification A (avoid) was not included as it does not list any antimicrobial substances licensed for use in food-producing animals).

### 5.4. Statistical Analyses

All statistical analyses were carried out using the statistical programming language R [44]. The data were prepared and plots were created using the tidyverse package [45].

## Figures and Tables

**Table 1:** Proportion in percent per year of antimicrobials dispensed for use in Austrian pigs for different farm types, based on mg/PCU.

Production System	2015	2016	2017	2018	2019	2020
Farrow-to-finish	34.1	34.5	35.6	35.5	35.1	35.3
Fattening	36.0	38.0	36.6	37.6	38.7	40.0
Piglet rearing	1.5	1.2	0.9	1.1	1.1	0.8
Breeding	23.9	23.6	23.9	22.2	22.4	23.1
Not assignable	4.6	2.7	3.1	3.7	2.8	0.8

**Table 2:** Proportion in percent per year of antimicrobial classes dispensed for use in Austrian pigs, based on the European Medicines Agency’s DDDvet.

Proportion of Antimicrobials Dispensed in % per Year						
Antimicrobial Class	2015	2016	2017	2018	2019	2020
Tetracyclines	44.68	45.33	46.57	50.43	39.77	43.79
Penicillins with extend. spectrum	13.72	13.88	15.43	14.02	15.54	15.22
Polymyxins	8.01	8.63	7.65	6.93	8.18	9.51
Macrolides	8.67	8.43	9.10	9.77	9.87	9.00
Aminoglycosides	1.46	1.20	1.21	3.66	10.83	8.30
Sulfonamides	3.99	3.94	4.72	4.24	4.34	3.55
Trimethoprim and derivatives	3.79	3.81	4.57	4.00	4.05	3.22
Fluoroquinolones	2.15	2.35	2.57	2.30	2.57	2.83
Pleuromutilins	0.94	1.17	0.98	1.42	1.58	1.53
3rd/4th-gen. cephalosporins	0.96	1.05	1.03	0.95	1.09	1.10
β-lactamase-sensitive penicillins	0.94	0.96	0.93	0.82	0.90	0.94
Amphenicols	0.43	0.41	0.38	0.44	0.42	0.43
Lincosamides	5.43	4.74	2.78	0.48	0.47	0.37
Other antibacterials	4.82	4.10	2.10	0.54	0.39	0.21

**Table 3:** Proportion in percent per year of antimicrobial classes dispensed for use in Austrian pigs, based on mg/PCU.

Proportion of Antimicrobials Dispensed in % per Year						
Antimicrobial Class	2015	2016	2017	2018	2019	2020
Tetracyclines	63.91	63.61	61.88	64.37	56.40	59.58
Penicillins with extend. spectrum	14.25	14.54	16.23	14.73	18.51	17.76
Macrolides	6.36	6.20	6.47	7.02	7.63	6.86
Sulfonamides	5.92	5.78	6.84	6.07	6.91	5.34
Polymyxins	3.13	3.45	3.02	2.77	3.67	4.19
Aminoglycosides	1.14	1.08	0.96	1.10	2.17	1.90
Pleuromutilins	0.62	0.78	0.64	0.86	1.13	1.08
Trimethoprim and derivatives	1.13	1.15	1.37	1.21	1.38	1.07
β-lactamase-sensitive penicillins	0.78	0.80	0.77	0.69	0.85	0.87
Fluoroquinolones	0.32	0.36	0.38	0.36	0.43	0.46
Amphenicols	0.28	0.27	0.24	0.29	0.31	0.31
3rd/4th-gen. cephalosporins	0.15	0.17	0.16	0.15	0.20	0.20
Lincosamides	0.84	0.76	0.46	0.13	0.17	0.19
Other antibacterials	1.19	1.06	0.58	0.26	0.23	0.19

**Table 4:** Categorisation of veterinary antimicrobials according to the European Medicines Agency.

European Medicines Agency Category			
A (“Avoid”)	B (“Restrict”)	C (“Caution”)	D (“Prudent use”)
Not authorised for veterinary use in the European Union	Critically important for human health	Alternatives exist in human medicine	First line treatments but only when medically necessary
Carbapenems Glycopeptides Drugs used solely to treat tuberculosis etc.	Cephalosporins (3rd & 4th generation) Polymyxins Fluoroquinolones	Aminoglycosides Aminopenicillins (in combination with beta-lactamase inhibitors) Cephalosporins (1st & 2nd generation) Amphenicols Lincosamides Pleuromutilins Macrolides Rifaximin	Aminopenicillins Beta-lactamase sensitive penicillins Beta-lactamase resistant penicillins Sulphonamides (& combinations, incl. trimethoprim) Tetracyclines Nitrofuran derivatives Spectinomycin Bacitracin Fusidic acid Metronidazole

Based on the EMA AMEG infographic [18].

## Data Availability

The data included in this analysis were collated as part of a mandatory national monitoring programme and are not freely available in the public domain.

## Supplementary Materials

The following are available online at https://www.mdpi.com/article/10.3390/antibiotics11020216/s1, Figure S1: Antimicrobial classes (in mg/PCU) dispensed, divided by route of administration over time.
