# Supplementary material for: National Monitoring of Veterinary-Dispensed Antimicrobials for Use on Pig Farms in Austria: 2015–2020
Source: Antibiotics (Basel). 2022 Feb 8;11(2):216. doi: 10.3390/antibiotics11020216 (PMC8868257; doi:10.3390/antibiotics11020216)
Supplement: Supplementary file 1 [file antibiotics-11-00216-s001.zip › antibiotics-1578126-Supplementary.pdf]

Supplementary Materials

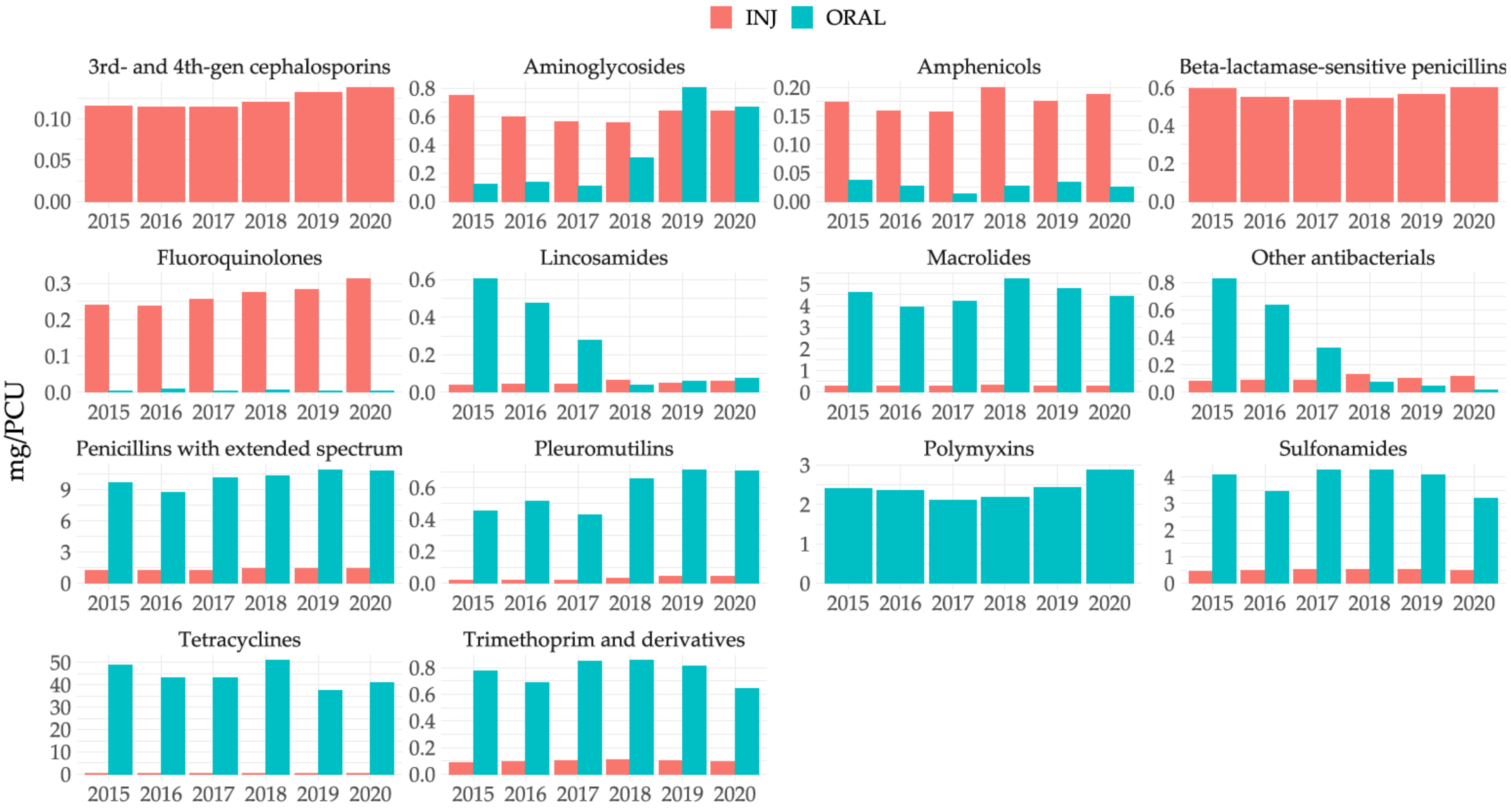

Figure S1. Antimicrobial classes (in mg/PCU) dispensed, divided by route of administration over time.
